# *Drosophila* Protamine-Like Mst35Ba and Mst35Bb Are Required for Proper Sperm Nuclear Morphology but Are Dispensable for Male Fertility

**DOI:** 10.1534/g3.114.012724

**Published:** 2014-09-17

**Authors:** Samantha Tirmarche, Shuhei Kimura, Laure Sapey-Triomphe, William Sullivan, Frédéric Landmann, Benjamin Loppin

**Affiliations:** * Centre de Génétique et de Physiologie Moléculaire et Cellulaire – CNRS UMR 5534 – Université Claude Bernard Lyon1, 69100 Villeurbanne, France; † Department of Molecular, Cell, and Developmental Biology, University of California Santa Cruz, Santa Cruz, California 95064; ‡ Centre de Recherche de Biochimie Macromoléculaire – CNRS UMR 5237 – 34293 Montpellier, France

**Keywords:** *Drosophila*, protamine-like, spermiogenesis, sperm, Mst35B

## Abstract

During spermiogenesis, histones are massively replaced with protamines. A previous report showed that *Drosophila* males homozygous for a genomic deletion covering several genes including the protamine-like genes *Mst35Ba/b* are surprisingly fertile. Here, we have precisely deleted the *Mst35B* locus by homologous recombination, and we confirm the dispensability of Mst35Ba/b for fertility.

In most animal species, the mature sperm nucleus is characterized by an extreme level of DNA compaction achieved after the massive replacement of somatic-type histones with sperm-specific nuclear basic proteins (SNBPs) (Lewis *et al.* 2003; Miller *et al.* 2010; Ward 2010; Kanippayoor *et al.* 2013). In mammals, the bulk of sperm chromatin is organized with two small protamines (Protamine 1 and 2) highly enriched in arginine residues (Balhorn 2007). *Drosophila* comprises at least three SNBPs: two paralogous protamine-like proteins, Mst35Ba and Mst35Bb, which are conserved among drosophilids, and the HILS1-related protein Mst77F (Russell and Kaiser 1993; Jayaramaiah Raja and Renkawitz-Pohl 2005; Alvi *et al.* 2013; Rathke *et al.* 2014). The almost identical Mst35Ba and Mst35Bb proteins are larger than mammalian protamines and are enriched in lysine residues. Mst35Ba/b proteins are incorporated in elongating spermatid nuclei at the late canoe stage and remain associated with mature sperm nuclei until fertilization (Jayaramaiah Raja and Renkawitz-Pohl 2005). Although the functions of *Drosophila* SNBPs remain poorly understood, Rathke *et al.* (2010) reported the surprising observation that *Drosophila* males homozygous for a genomic deficiency covering the *Mst35B* locus were fertile. This result was indeed unexpected when considering for instance the haploinsufficiency of mouse protamine genes for male fertility (Cho *et al.* 2001). However, according to Flybase (Flybase.org), the deficiency generated by Rathke *et al.* (2010) (named *protΔ*) is a 73.6-kb deletion that not only uncovers *Mst35Ba* and *Mst35Bb* but also removes four additional protein encoding genes (*CG42682*, *CG15279*, *CG4480*, *CG15278*) as well as three noncoding RNAs (*CR43805*, *CR45727*, *CR45302*). Furthermore, all these genes and noncoding RNAs are expressed in the adult testis or accessory glands, with the exception of *CG15279*, and transcripts of three of these genes (*CG33309*, *CG4480*, and *CG15278*) were detected in early spermatids (Flybase; Rathke *et al.* 2010). Because the simultaneous deletion of these other genetic elements could potentially interfere with a detailed functional analysis of *Mst35B* genes, we generated a precise deletion of the *Mst35B* locus by homologous recombination using the “Ends-Out” targeting technique (Gong and Golic 2003, 2004) (Figure 1A). The resulting allele, named *ΔMst35B*, eliminates a 5-kb genomic DNA fragment that only contains the *Mst35Ba* and *Mst35Bb* genes. To validate the elimination of these genes in the new deletion allele, we raised an antiserum against a peptide common to Mst35Ba and Mst35Bb proteins (Figure 1B). This antibody specifically stained late canoe stage spermatid nuclei of wild-type males but not those of *ΔMst35B* homozygous males (Figure 1C). At later stages of spermiogenesis, the highly compacted chromatin of spermatids is no longer accessible to antibodies (Bonnefoy *et al.* 2007), thus explaining the absence of staining beyond the canoe stage in wild-type testes. In addition, another anti-Mst35B antibody raised against the whole Mst35Bb recombinant protein allowed us to confirm the absence of Mst35B proteins from *ΔMst35B* testicular protein extracts (Figure 1D).

**Figure 1:**
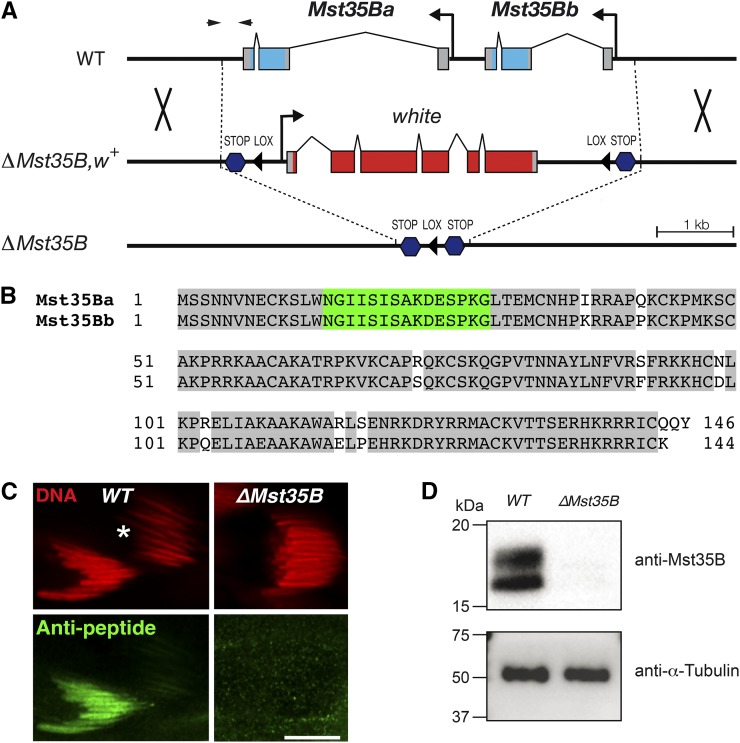
Knocking-out *Mst35Ba/b* genes by homologous recombination. (A) Representation of the *Mst35b* locus before (wild-type; *WT*) and after (*ΔMst35B*, *w+*) “ends-out” targeting using the *pW25* vector (Gong and Golic, 2003, 2004; details are available upon request). The *ΔMst35B* allele was obtained after *Cre*-mediated excision of the *white* cassette. The tandem protamine genes *Mst35Ba* and *Mst35Bb* are represented with their coding regions in pale blue. The dark blue hexagons representing termination codons in the six reading frames and the Lox sites (black triangles) are from the *pW25* ends-out targeting vector. Arrowheads indicate the position of the primer pair used in polymerase chain reaction analyses. (B) Sequence alignment of Mst35Ba and Mst35Bb proteins. The peptide used to generate the rabbit antipeptide “NGI” polyclonal antibody is highlighted in green and other identical residues are shaded in gray. (C) Immunofluorescence staining of *WT* or *ΔMst35B* spermatid nuclei with the antipeptide “NGI” antibody. The antibody stains *WT* spermatid nuclei in late canoe stage but not more advanced nuclei (asterisk), which are too compacted for antibody penetration (Bonnefoy *et al.* 2007). No staining was observed in mutant spermatid nuclei at the late canoe stage. Bar: 10 µm. (D) Western blot analysis of testicular protein extracts using a rabbit antibody directed against a recombinant full-length Mst35Bb protein. Mst35Ba/b-specific bands are detected in WT but not in *ΔMst35B* extracts. The anti-α-Tubulin antibody was used as loading control.

As expected, homozygous *ΔMst35B* males were fully viable (not shown) and at least partially fertile (see paragraphs to follow), thus confirming the dispensability of Mst35B proteins for male fertility. Spermiogenesis (the differentiation of postmeiotic spermatids) in mutant males nevertheless appeared severely disorganized, with many elongating spermatids showing abnormal nuclear morphology (Figure 2, A−C). The spermiogenesis defects were similar in homozygous *ΔMst35B* and *trans*-heterozygous *ΔMst35B/protΔ* males, ruling out the possibility that the phenotypes associated with *ΔMst35B* were caused by a second-site mutation. In both allelic combinations, affected spermatid nuclei typically appeared bent compared with control spermatids, with the anterior tip of the nucleus sometimes folded into a hook-like structure (Figure 2, D and E). It is likely that the concentration of chromatin at one end of mutant spermatid nuclei observed by Rathke *et al.* (2010) actually correspond to folded nuclear extremities. A large proportion of mutant spermatids were scattered along the cysts instead of remaining tightly grouped in bundles of 64 nuclei, suggesting that they were progressively eliminated during the course of spermiogenesis (Figure 2, B and C). Accordingly, mutant males stored significantly less gametes in their seminal vesicles compared with control males (Figure 3E). Interestingly, however, we did observe morphologically aberrant mature gametes stored in the seminal vesicles of homozygous *ΔMst35B* and *ΔMst35B/protΔ* males (Figure 3, A−C), in sharp contrast to previous observations (Rathke *et al.* 2010). A transgene expressing Mst35Ba-EGFP rescued the abnormal nuclear shaping of *ΔMst35B* spermatids, thus confirming that this phenotype is actually caused by the loss of *Mst35B* genes (Figure 3D). However, a fraction of spermatids was still eliminated in rescued animals (Supporting Information, Figure S1), suggesting that the presence of a relatively large green fluorescent protein tag perturbs the functionality of the recombinant protein. Alternatively, both Mst35Ba and Mst35Bb proteins could be required for proper packaging of sperm DNA. In addition, we confirmed that a transgene expressing Mst77F-EGFP was normally incorporated into the chromatin of mutant gametes but failed to rescue the phenotype (Figure 3B). Finally, using a specific antibody (Figure S2), we also verified that the transition protein Tpl94D (Rathke *et al.* 2007) was normally incorporated in mutant spermatids at the histone-to-protamine transition (Figure 2, D and E), confirming that the nuclear defects in mutant spermatids appear after this stage.

**Figure 2:**
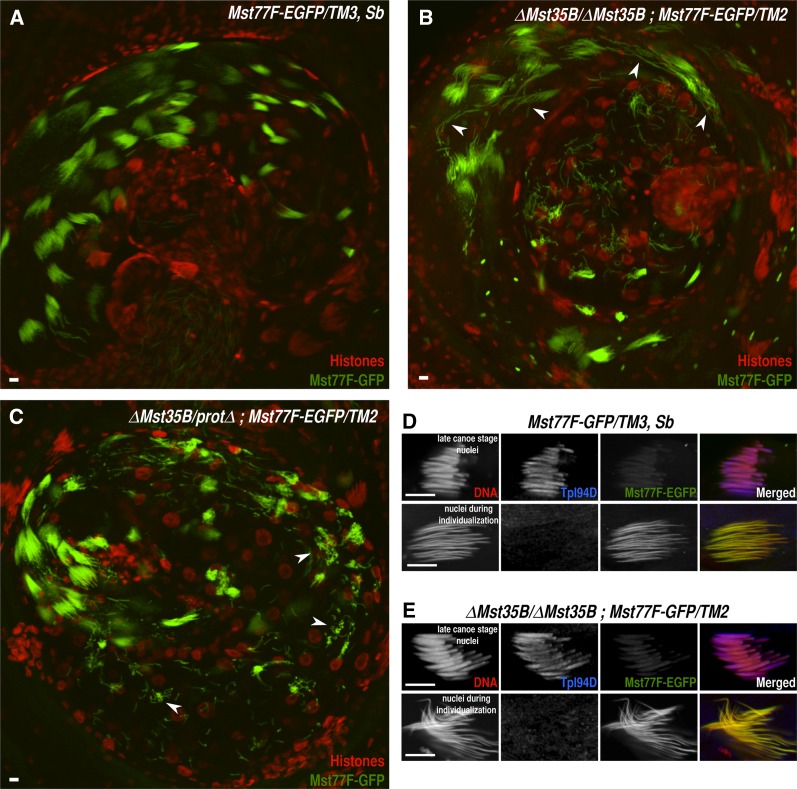
Late spermiogenesis defects of *ΔMst35B* males. (A−C) Confocal images of control (A), *ΔMst35B* (B), or *ΔMst35B*/*protΔ* (C) testes expressing a *Mst77F-EGFP* transgene and stained with an anti-histone antibody (MABE71; Millipore) in red. In control testes, groups of 64 spermatid nuclei are visible in each cyst. Needle-like shaped spermatid nuclei appear green after the removal of histones and the incorporation of Mst77F-EGFP. In *ΔMst35B*/*ΔMst35B* as in *ΔMst35B*/*protΔ* testes, many green fluorescent protein− positive spermatid nuclei appear scattered along the length of the cyst (arrowheads), indicating that they are progressively eliminated. (D−E) Confocal images of spermatid nuclei from control (D) and protamine mutant testes (E) bearing a *Mst77F-EGFP* transgene. Testes were immunostained for Tpl94D, a transition protein facilitating the deposition of sperm-specific nuclear basic proteins (Rathke *et al.* 2007). The anti-Tpl94D antibody was generated by rabbit immunization with full-length recombinant Tpl94D protein (details available upon request. See Figure S2). In both control and *ΔMst35B* mutant testes, spermatid nuclei successively incorporate Tpl94D and Mst77F-EGFP. However, mutant spermatids frequently exhibit a twisted shape compared to control nuclei. Scale bar: 10 μm

**Figure 3:**
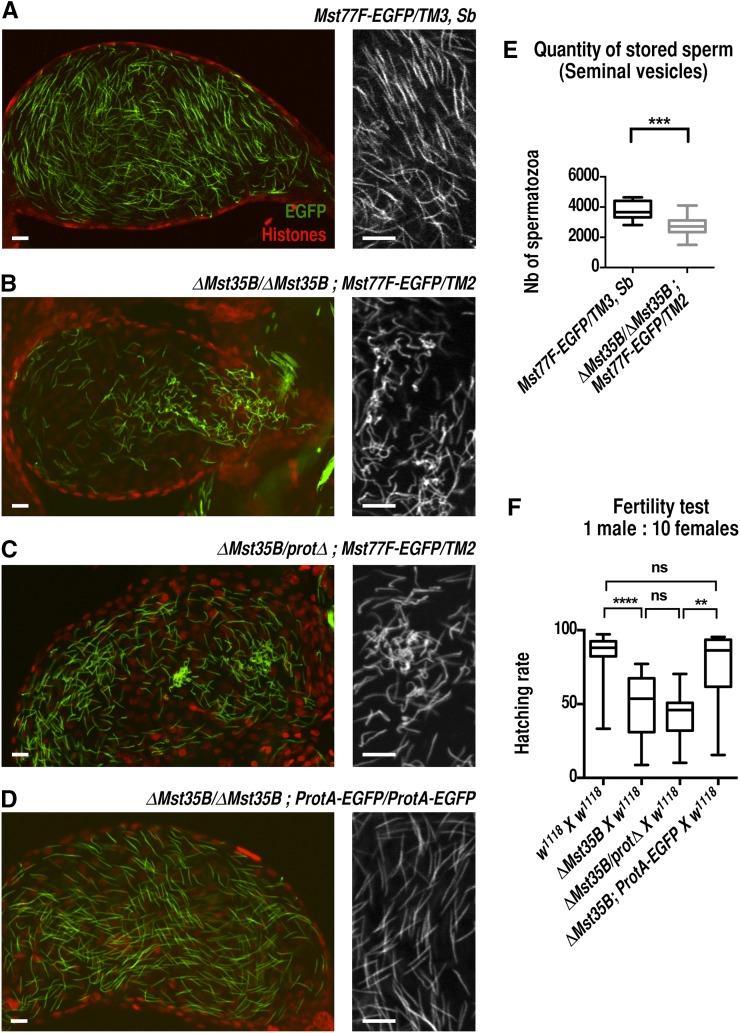
Male sperm storage and fertility is affected by *ΔMst35B* deletion. (A−D) Confocal images of seminal vesicles of the indicated genotype stained with an anti-histone antibody (red) that reveals somatic nuclei. Sperm nuclei (green) are labeled with Mst77F-EGFP (A-C) or ProtA-EGFP (D). Magnified views are shown in gray. Seminal vesicles of *ΔMst35B/ΔMst35B* (B) and *ΔMst35B/protΔ* (C) mutant males contain abnormally shaped sperm nuclei compared with straight nuclei observed in control (A) or rescued (D) males. Scale bar: 10 μm. (E) Quantification of sperm nuclei in seminal vesicles dissected from four days old virgin males. Images of squashed seminal vesicles labeled as in (A−B) were acquired with a Z1 Imager microscope (Zeiss) and sperm nuclei were individually counted using the Image J software. A total of 9 control vesicles and 14 *ΔMst35B* mutant vesicles were counted and the results were statistically analyzed with a Mann-Whitney test. ***P < 0.001. (F) Reduced fertility of *ΔMst35B* males. 0- to 1-d-old males of the indicated genotypes were individually mated with 10 virgin *w^1118^* females. Embryo hatching rates were then determined as described in Orsi *et al.* (2010). Results were statistically analyzed with a Mann-Whitney *U* test. *ns*: nonsignificant. **P < 0.01. ****P < 0.0001.

Although the quantity and quality of gametes were affected by the loss of *Mst35B* genes, homozygous *ΔMst35B* and *ΔMst35B/protΔ* males were nevertheless fertile, in agreement with the study by Rathke *et al.* (2010). In fact, the impact of *ΔMst35B* on male fertility was only revealed when mutant males were allowed to mate with a large excess of virgin females (1 for 10; Figure 3F) but not with a 1:1 sex ratio (not shown). In the presence of a large excess of females, the observed reduction of fertility is likely explained by the limiting amount of sperm produced by mutant males (Figure 3E).

The organization of sperm chromatin in animals is poorly understood and most of our knowledge comes from studies on human or other mammalian species. *Drosophila* is an interesting, alternative model for the study of sperm chromatin at the functional level. The generation of a precise deletion allele of both protamine-like genes *Mst35Ba/b* provides an ideal tool for the functional study of *Drosophila* SNBPs. The fertility of *ΔMst35B* males reveals the extraordinary plasticity of the *Drosophila* sperm nucleus, which grossly maintains its architecture, motility and ability to fertilize eggs in the absence of what is considered a major component of its chromatin. It is likely that additional SNBPs compensate for the loss of the protamine-like proteins. In fact, we already know that the loss of Mst35Ba/b proteins does not perturb the incorporation of Mst77F in spermatid nuclei (this work and Rathke *et al.* 2010). *Mst77F*, which was originally identified in a genetic screen for β2 tubulin interactors (Fuller *et al.* 1989), is related to the mammalian spermatid-specific histone H1-like protein HILS1 (Iguchi *et al.* 2004; Yan *et al.* 2003). Interestingly, the *D. melanogaster* genome contains several recent copies of *Mst77F* on the Y chromosome, and eight of these *Mst77Y* genes are most likely functional (Russell and Kaiser 1993; Krsticevic *et al.* 2010). It has been proposed that Mst77F is essential for male fertility (Rathke *et al.* 2010), but this conclusion is based on the analysis of the antimorphic point mutation *Mst77F^1^* (see Krsticevic *et al.* 2010). Future work should aim at clarifying the nuclear function of Mst77F/Y proteins to determine if they can indeed maintain a sperm chromatin structure compatible with male fertility in the absence of Mst35B proteins.

## Supplementary Material

Supporting Information
