# Supplementary material for: Drosophila Protamine-Like Mst35Ba and Mst35Bb Are Required for Proper Sperm Nuclear Morphology but Are Dispensable for Male Fertility
Source: G3 (Bethesda). 2014 Sep 17;4(11):2241–5. doi: 10.1534/g3.114.012724 (PMC4232549; doi:10.1534/g3.114.012724)
Supplement: Supporting Information [file supp_4_11_2241__index.html]

Drosophila Protamine-Like Mst35Ba and Mst35Bb Are Required for Proper Sperm Nuclear Morphology but Are Dispensable for Male Fertility — Supporting Information 

# *Drosophila* Protamine-Like Mst35Ba and Mst35Bb Are Required for Proper Sperm Nuclear Morphology but Are Dispensable for Male Fertility

## Supporting Information for Tirmarche *et al.*, 2014

**Files in this Data Supplement:**

- Supporting Information - Figures S1-S2 (PDF, 806 KB)
- Figure S1 - Confocal image of a homozygous *ΔMst35B* testis expressing a *Mst35Ba-GFP* transgene and stained with an anti-histone antibody (Millipore, MABE71) in red. (PDF, 368 KB)
- Figure S2 - Confocal images of spermatid nuclei from *WT* testes expressing a *Tpl94D-RFP1* transgene. (PDF, 497 KB)
