# Supplementary material for: Drosophila Protamine-Like Mst35Ba and Mst35Bb Are Required for Proper Sperm Nuclear Morphology but Are Dispensable for Male Fertility
Source: G3 (Bethesda). 2014 Sep 17;4(11):2241–5. doi: 10.1534/g3.114.012724 (PMC4232549; doi:10.1534/g3.114.012724)
Supplement: Supporting Information [file supp_g3.114.012724_012724SI.pdf]

G3·Genes | Genomes | Genetics

Drosophila protamine-like Mst35Ba and Mst35Bb are required for proper sperm nuclear morphology but are dispensable for male fertility

Samantha Tirmarche*, Shuhei Kimura*, Laure Sapey-Triomphe*, William Sullivan†, Frédéric Landmann‡ and Benjamin Loppin*,§

* Centre de Génétique et de Physiologie Moléculaire et Cellulaire – CNRS UMR 5534 – Université Claude Bernard Lyon1, 69100 Villeurbanne, France.
† Department of Molecular, Cell, and Developmental Biology, University of California Santa Cruz, Santa Cruz, CA 95064, USA.
‡ Centre de Recherche de Biochimie Macromoléculaire – CNRS UMR 5237 – 34293 Montpellier, France.
§ Corresponding author : benjamin.loppin@univ-lyon1.fr

Corresponding author:

Benjamin Loppin
Centre de Génétique et de Physiologie Moléculaire et Cellulaire – CNRS UMR 5534 – 16 rue Raphaël Dubois, Bât. Mendel, Université Claude Bernard Lyon1, 69100 Villeurbanne, France.
Phone: 0033 472 447 926
Email: benjamin.loppin@univ-lyon1.fr

DOI: 10.1534/g3.114.012724

Tirmache *et al.* Figure S1

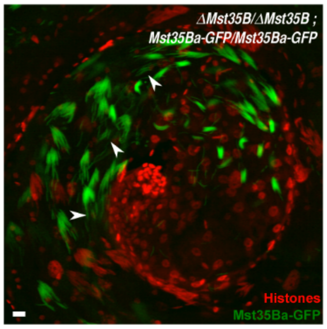

Figure S1 Confocal image of a homozygous *ΔMst35B* testis expressing a *Mst35Ba-GFP* transgene and stained with an anti-histone antibody (Millipore, MABE71) in red. Arrowheads point to eliminated spermatids. Scale bar: 10 µm.

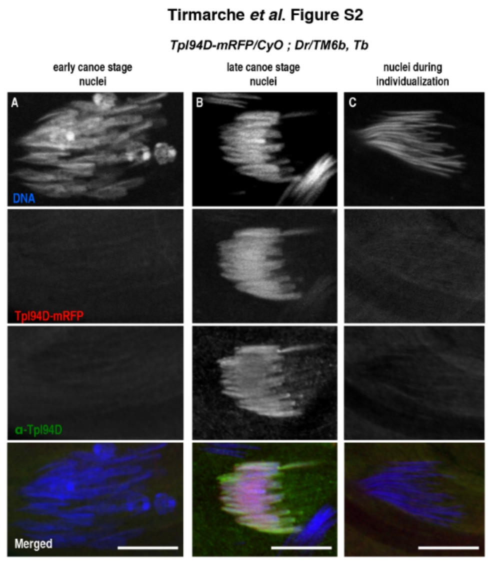

Figure S2 Confocal images of spermatid nuclei from *WT* testes expressing a *Tpl94D-RFP1* transgene. Testes were stained with an anti-Tpl94D (green) and Tpl94D-mRFP1 was observed through its native fluorescence (red). Scale bar: 10 µm
