# Supplementary material for: Drosophila Protamine-Like Mst35Ba and Mst35Bb Are Required for Proper Sperm Nuclear Morphology but Are Dispensable for Male Fertility
Source: G3 (Bethesda). 2014 Sep 17;4(11):2241–5. doi: 10.1534/g3.114.012724 (PMC4232549; doi:10.1534/g3.114.012724)
Supplement: Supporting Information [file supp_g3.114.012724_FigureS1.pdf]

Tirmache *et al.* Figure S1

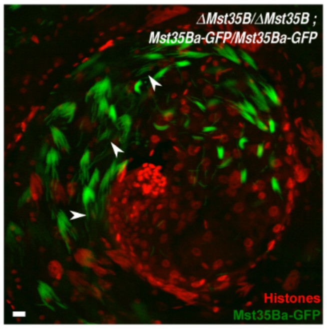

Figure S1 Confocal image of a homozygous *ΔMst35B* testis expressing a *Mst35Ba-GFP* transgene and stained with an anti-histone antibody (Millipore, MABE71) in red. Arrowheads point to eliminated spermatids. Scale bar: 10 µm.
