# Supplementary material for: Drosophila Protamine-Like Mst35Ba and Mst35Bb Are Required for Proper Sperm Nuclear Morphology but Are Dispensable for Male Fertility
Source: G3 (Bethesda). 2014 Sep 17;4(11):2241–5. doi: 10.1534/g3.114.012724 (PMC4232549; doi:10.1534/g3.114.012724)
Supplement: Supporting Information [file supp_g3.114.012724_FigureS2.pdf]

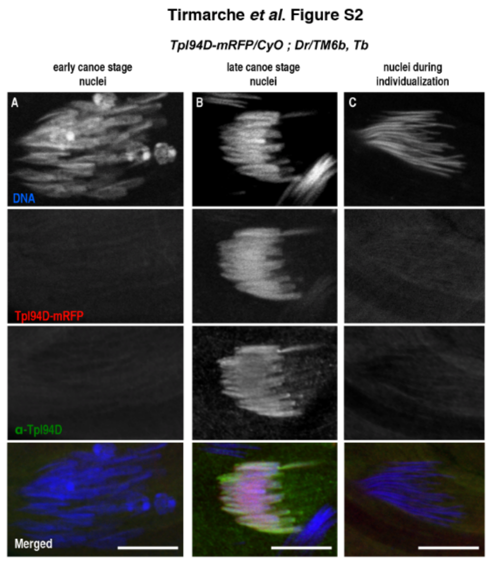

Figure S2 Confocal images of spermatid nuclei from *WT* testes expressing a *Tpl94D-RFP1* transgene. Testes were stained with an anti-Tpl94D (green) and Tpl94D-mRFP1 was observed through its native fluorescence (red). Scale bar: 10 µm
